# Repurposing Eltrombopag as an Antimicrobial Agent Against Methicillin-Resistant *Staphylococcus aureus*

**DOI:** 10.3389/fmicb.2021.790686

**Published:** 2022-01-24

**Authors:** Pengfei She, Shijia Li, Linying Zhou, Yaqian Liu, Lanlan Xu, Zubair Hussain, Yimin Li, Zehao Li, Shasha Liu, Yong Wu

**Affiliations:** ^1^Department of Laboratory Medicine, Third Xiangya Hospital of Central South University, Changsha, China; ^2^Department of Laboratory Medicine, The First Hospital of Changsha, Changsha, China

**Keywords:** eltrombopag, drug repurposing, methicillin-resistant *Staphylococcus aureus*, biofilm, *in vivo*

## Abstract

Because of the excessive use of antibiotics, methicillin-resistant *Staphylococcus aureus* (MRSA) has become prevalent worldwide. Moreover, the formation of *S. aureus* biofilms often cause persistence and relapse of infections. Thus, the discovery of antibiotics with excellent antimicrobial and anti-biofilm activities is urgently needed. In the present study, eltrombopag (EP), a classic thrombopoietin receptor agonist, exhibited potential antimicrobial activity against *S. aureus* and its biofilms. Through our mechanistic studies, EP was found to interfere with proton motive force in *S. aureus*. The *in vivo* anti-infective efficacy of EP was further confirmed in the wound infection model, thigh infection model and peritonitis model by MRSA infection. In addition, the cytotoxicity of EP against mammalian cells and the *in vivo* toxicity of EP in animal models were not observed at the tested concentrations. Collectively, these results indicate that EP could be considered a potential novel antimicrobial agent against recalcitrant infections caused by MRSA.

## Introduction

*Staphylococcus aureus* is the main cause of hospital- and community-acquired infections, which can cause various infectious diseases, including bacteremia, infective endocarditis, osteomyelitis, septic arthritis, sepsis, and lethal pneumonia (Lakhundi and Zhang, 2018). Because of the excessive use of antibiotics, methicillin-resistant *S. aureus* (MRSA) has become prevalent worldwide. MRSA, including hospital-acquired MRSA (HA-MRSA) and community-acquired MRSA (CA-MRSA), has rapidly become one of the most frequently occurring resistant pathogen in many countries (Lakhundi and Zhang, 2018; Guo et al., 2020). MRSA accounts for 13%–74% of global *S. aureus* infections (Morroni et al., 2018). Based on the China Antimicrobial Surveillance Network database, *S. aureus* is one of the most frequent isolated Gram-positive pathogen in China, with HA-MRSA accounting for 50.4% of these isolates (Lee et al., 2018). In another study, the resistant percentages of *S. aureus* to gentamicin and rifampicin (RFP) were 24.1% and 12%, respectively (Hassoun et al., 2017; Lee et al., 2018). Thus, it is indisputable that clinical treatment targeting *S. aureus* infections is a tremendously formidable and imperative task.

*Staphylococcus aureus* biofilms, which are commonly found on implantable medical devices such as contact lenses, urinary and central venous catheters, and prosthetic joints, are the most frequent cause of refractory infections. These biofilms protect the bacterial cells from antibiotic attack (Raafat et al., 2019). A biofilm is an aggregation of cells surrounded by an exopolymeric matrix. The biofilms exhibit high resistance to attacks by phagocytes and antibiotics due to impairment in penetrating through the biofilm matrix (Otto, 2018). The primary resistance mechanism of *S. aureus* biofilm is immune evasion by impeding phagocytic function and appropriate anti-inflammatory responses (Bhattacharya et al., 2018). However, currently, no antibiotic is present that can effectively eradicate these biofilms.

Many challenges in the development of novel antibiotics remain to be overcome. In particular, high research costs, low investment returns, and strict regulations have resulted in a scarcity of research on novel antibiotics (Piddock, 2012). Moreover, drug development is arduous and time-consuming. For this problem, drug repurposing, which is the identification of new therapeutic uses for approved drugs, is considered a viable strategy (Ashburn and Thor, 2004). This process involves the use of drugs that are familiar to clinicians, generates detailed safety data, and has short research cycles, low developmental costs, and substantial benefits. It can also provide information on new antibacterial targets and the molecular mechanisms of the antibacterial effects of the drugs.

Eltrombopag (EP), a non-peptide small molecule agonist of the human thrombopoietin receptor (TpoR), was discovered by screening small molecular libraries to bind TpoR and stimulate its downstream signaling, and further rationally designed to improve yielding and purity (Bussel et al., 2019). The bisolamine salt EP Olamine [EP(O)] is the most common formulation, which was found to be easily produced, orally ingestible and environmentally stable (Abbinante et al., 2020). EP is mainly used in the oral treatment of idiopathic thrombocytopenic purpura (ITP) (Erickson-Miller et al., 2009). It binds to the transmembrane domain of TpoR (c-Mpl) on bone marrow megakaryocytes, activates JAK/STAT and MAPK signaling pathways, and stimulates platelet production (Di Buduo et al., 2016). EP has also been widely studied in the treatment of other diseases. For example, a recent study confirmed using high-throughput molecular docking technology and *in vitro* phenotypic screening tests that EP has antibacterial effects against *Mycobacterium tuberculosis* possibly *via* the inhibition of *M. tuberculosis* proteins, Zmp1 and peptide deformylase (Battah et al., 2019). EP has a limited spectrum of antifungal activity against *Cryptococcus neoformans*, as it affects the pathogenicity of *C. neoformans*, particularly in terms of capsule and biofilm formation, melanin production, and ability to grow at 37°C (Ko et al., 2020). EP was also found to inhibits DNA replication in Ewing sarcoma cells and human cytomegalovirus *via* iron chelation (Vogel et al., 2019; Waters et al., 2020). In addition, our previous study reported that EP showed effective *in vitro* antimicrobial effects against *Staphylococcus epidermidis* with MIC and MBC of 8 and 16 μg/mL, respectively (Zhu et al., 2021). However, to the best of our knowledge, studies pertaining to the anti-*S. aureus* activity of EP have not been performed.

In the present study, we investigated the antimicrobial activity of EP against MRSA and its biofilms, and determined the roles of the disruption of the proton motive force (PMF) in the antimicrobial mechanism of EP. We further assessed the antimicrobial activity of EP *in vivo* using animal models. In addition, although the toxic profile of EP was well studied as a TpoR agonist, the *in vivo* toxicity of EP as an antimicrobial was further determined in our study because the different *in vivo* administration routes, doses and periods of EP were needed for different purposes.

## Materials and Methods

### Strains and Culture Conditions

The type strains of *S. aureus* ATCC 25923, ATCC 29213, and *Enterococcus faecalis* ATCC 29212 were provided by Juncai Luo (Tiandiren Biotech, Changsha, China). While type strains *S. aureus* ATCC 43300, USA300, and MW2 were donated by Min Li (Renji Hospital, School of Medicine, Shanghai Jiao Tong University). Moreover, *S. epidermidis* type strains RP62A and ATCC 12228 were obtained from Di Qu (Laboratory of Medical Molecular Virology, Shanghai Medical College, Fudan University, Shanghai, China). *Pseudomonas aeruginosa* type strain PAO1 was provided by Qiao Minqiang (College of Life Sciences of Nankai University, Tianjin, China). Clinical strains of *S. aureus* and *Enterococcus faecium* were isolated from the Third Xiangya Hospital of Central South University, and identified by VITEK 2 Compact (bioMerieux, France) and Matrix-Assisted Laser Desorption Ionization (BD, Germany). *S. aureus* and *S. epidermidis* were grown in Tryptic Soy Broth (TSB) (Solarbio, Beijing, China), *E. faecalis* and *E. faecium* were grown in brain heart infusion (BHI, Solarbio, Beijing, China) broth. Unless specifically mentioned, all the work on MRSA pertains to the type strain USA300.

### Antimicrobial Agents and Chemicals

EP, EP(O), RFP, vancomycin (VAN), ciprofloxacin (CIP) and Killophor were purchased from MedChemExpress (Shanghai, China). We prepared 15-20 mg/mL stock solutions of all compounds in dimethyl sulfoxide (DMSO, Sigma-Aldrich, Shanghai, China) or ddH_2_O (TansGen Biotech, Beijing, China).

### Antimicrobial Susceptibility Test

The antimicrobial activity was evaluated by broth microdilution method recommended by the Clinical and Laboratory Standards Institute (CLSI, 2020). Briefly, the overnight culture of bacterial suspension was adjusted to 0.5 McFarland standard in Mueller-Hinton (MH) broth (RiShui Bio-tech, Qingdao, China). The bacterial suspension (approximately 1.5 × 10^6^CFU/mL) was mixed with serially diluted drugs in 96-well plate and incubated at 37°C for 16–18 h. And the concentration at which no bacterial growth was visible to the naked eye was defined as the minimal inhibitory concentration (MIC). Furthermore, the concentration that killed 99.9% of bacterial colonies streaked on 5% sheep blood agar plates (Autobio, Zhengzhou, China) was defined as the minimal bactericidal concentration (MBC). These experiments were performed in triplicate.

### Time-Killing Assay

A single colony of *S. aureus* was inoculated into 10 mL of TSB medium and cultured overnight at 37°C with shaking at 150 rpm. Then the bacterial suspension was diluted with EP at 0.5 × MIC, 1 × MIC, 2 × MIC and 4 × MIC to a final concentration of 1 × 10^6^CFU/mL, and 0.5% DMSO TSB was used as a control. The samples were collected at the time point of 0, 2, 4, 8, 12, and 24 h, 10-fold serially diluted in saline, and 100 μL of the bacterial suspension was spread on a sheep blood agar plate. After incubation at 37°C for 24 h, the colonies were counted in colony forming units per milliliter (CFU/mL). All experiments were conducted in triplicate.

### Checkerboard Assay

Firstly, we determine the MIC of each drugs as described above. Next, the mid-log-phase bacterial suspension was adjusted to 5 × 10^5^ CFU/mL, and 50 μL of the suspension was added to each well in a 96-well plate to form an 8 × 8 “checkerboard.” Twenty-five microliter of the two diluted drugs were added to 96-well plates horizontally and longitudinally to ensure that different concentrations of the two drugs were mixed in each well. After incubation at 37°C for 18 to 24 h, the optical density at 630 nm (OD630) was measured. The fractional inhibitory concentration index (FICI) was calculated as FICI = MIC_A_ combined/MIC_A_ alone + MIC_B_ combined/MIC_B_ alone, FIC ≤ 0.5 indicates synergy, 0.5 < FIC ≤ 1 indicates partial synergy, and FIC > 1 indicates no interaction (She et al., 2019). All experiments were conducted at least in duplicate.

### Resistance Inducing Assay

To induce resistant mutants of *S. aureus* by EP, the MICs of EP against *S. aureus* ATCC 43300, USA300 and MW2 was detected by the MH broth micro-dilution method as described above. Then, the bacterial suspension in the sub-inhibitory concentration well (1/2 × MIC) was diluted 1000 times to prepare for the next day’s MIC test. The process of drug resistance induction lasted for 16 days, and the change of MIC value was recorded (Kim et al., 2018).

### Single-Step Resistance Selection

An overnight culture of *S. aureus* USA300 was sub-cultured to log-phase in fresh TSB broth, and was adjusted to an OD630 of 0.5. Then, 100 μL of the diluted bacterial cultures was spread on MH agar in the presence of 2 × MIC, 4 × MIC of EP or RFP as comparative control. Meanwhile, the starting inoculum was quantified by CFU countings. After incubation at 37°C for 48 h, the resistance frequency was calculated as the number of drug-resistant mutants divided by the number of total colonies (Trombetta et al., 2018).

### Biofilm Formation Inhibition Assay

The overnight culture of *S. aureus* was diluted at 1: 100 with TSB in the presence of 0.15% glucose (TSB-g). Then a 50 μL diluted suspension was mixed with an equal amount of TSB-g containing two-fold diluted EP (0∼16 μg/mL). After incubation at 37°C for 24 h, the non-adherent cells were removed by washing with PBS three times. The plates were air-dried at room temperature, and the OD630 was determined as the biomass of biofilm (She et al., 2019). All experiments were conducted in triplicate.

### Biofilm Eradication Assay

The overnight culture of *S. aureus* was diluted at 1: 200 with TSB-g, and 200 μL of the suspension was added to each well of a 96-well cell culture plate. The plate was statically incubated at 37°C for 24 h and washed 3 times with PBS to remove non-adherent cells. Then, 200 μL of two-fold diluted VAN (0∼64 μg/mL) or EP (0∼32 μg/mL) were added to each well. After incubation for 24 h, the plates were washed with PBS, and the residual biofilm was detected by OD630 (She et al., 2019). All experiments were conducted in triplicate.

### Membrane Disruption Determination by Fluorescent Dyes

The membrane disruption was detected by SYTOX Green and DiSC3(5) staining. In short, the mid-log phase bacterial suspension was adjusted to OD630 = 0.05 with 5 mmol/L HEPES (pH7.4). For SYTOX Green staining, the above suspension was added with 2 μmol/L SYTOX Green in the dark for 15 min, and then incubated with different concentrations of EP (0–4 × MIC), 0.1% DMSO (negative control) or 10 μg/mL otilonium bromide (OB, positive control). The fluorescence intensity was recorded by a microplate reader (PerkinElmer EnVision, United States) every 5 min for a total of 30 min (excitation/emission wavelength = 504/523 nm). For DiSC3(5) staining, the above-mentioned suspension was treated in the dark with 5 mmol/L glucose, 100 mmol/L KCl and 2 μmol/L Disc3(5) for 1 h, and the fluorescence intensity was monitored with the microplate reader every 30 s for 5 min (excitation/emission wavelength = 622/670 nm) (Zhou et al., 2020). All experiments were conducted in triplicate.

### Effects of pH-Adjusted Proton Motive Force on Antimicrobial Activity of Eltrombopag

The pH value of pH-adjusted media was ranging from 4.0 to 9.0 and prepared by addition of HCl or NaOH into the MH broth. *S. aureus* USA300 was cultured overnight at 37°C, diluted 1: 10,000 with fresh MH broth, and sub-cultured to mid-log phase. The suspension was then diluted to a final concentration of 1 × 10^6^ CFU/mL with a pH-adjusted media containing the specified concentration of EP (0–24 μg/mL). Then 100 μL of the diluted cells were added to a 96-well plate and incubated at 37°C for 16 h, and the growth turbidity of the bacterial suspension was measured by OD630 (Bhando et al., 2020). The experiment was conducted triplicate.

### Acute Wound Infection Model

All experimental procedures were performed on animals according to the guidelines of the IRB of the Third Xiangya Hospital of Central South University. Eight-week-old BALB/c mice were purchased from Hunan SJA Experimental Animal Co. Ltd. (Changsha, China). The model of neutropenia was established by treating with cyclophosphamide 150 mg/kg (day – 4) and 100 mg/kg (day – 1). The mice were anesthetized with isofluorane (i.p.), their backs were shaved and disinfected with 75% ethanol. A 1.5 cm^2^ wound to the basal layer of epidermis was made by scratching using a needle (4.5 to 5 #). Then ∼ 2.5 × 10^5^ CFU of *S. aureus* USA300 with 30 μL of PBS was inoculated on the surface of the artificial wound. After 1 h of the inoculation, ointments (vehicle) alone or ointments containing EP (2% w/v) was gently applied with swabs at intervals of 5 h for 20 h. After 4 h of the last treatment, mice were euthanized and the wounded tissue were collected, homogenized and quantified by serial dilution method (Stokes et al., 2020).

### Peritonitis-Sepsis Model

Six-week-old female ICR mice with an average weight of 25 ± 3 g, were selected for *in vivo* experiments. Mice were purchased from Hunan SJA Experimental Animal Co. Ltd. (Changsha, China). The mouse peritonitis model was performed as previously described by Su et al. (2019) with slight modifications. Overnight bacterial cultures of *S. aureus* USA300 were diluted to 5 × 10^6^ CFU/mL with PBS. Then 500 μl of suspension containing 5% (w/v) mucin were injected to mice *via* i.p. One hour after infection, mice were treated with 2 doses of EP (30 mg/kg, i.p.) at 12 h interval or 4 doses of VAN (30 mg/kg, i.p.) at an interval of 5 h alone, or the combination of EP and VAN. 5% Killophor + 5% ethanol was used as vechile. The mice were euthanized after 24 h of the first dose and the spleen and liver were excised and homogenized in 1 mL of PBS, then the bacterial count was quantified by serial dilution (Ikeh et al., 2018).

### Neutropenic Murine Thigh Infection Model

All mice were rendered neutropenic by treatment with cyclophosphamide on day – 4 (150 mg/kg) and day – 1 (100 mg/kg) prior to bacterial infection. To induce thigh deep infection, overnight culture of *S. aureus* USA300 was adjusted to 5 × 10^6^ CFU/mL in PBS. Then 50 μL of the suspension was injected intramuscularly in the right thigh muscle of the mice. Therapy was initiated after 1 h post-infection. One milliliter of EP (30 mg/kg, i.p. two doses at an interval of 12 h) or 200 μL of VAN (30 mg/kg, i.p. 4 doses at an interval of 5 h) alone or in combination were used to treat the infected mice, respectively. 5% Killophor + 5% ethanol was used as a vehicle control. Mice were euthanized 24 h after the first dose, and the thighs were excised and homogenized in 2 mL of PBS. The homogenates were used for the determination of the bacterial count after inoculation on sheep blood agar plates (Kim et al., 2019; Su et al., 2019). All the experiments were performed at least triplicate.

### Statistical Analyses

All analyses were conducted three times unless otherwise stated. Statistical analysis was performed using Graph-Pad Prism 8.0 software and all data were expressed as means ± S.D./S.E. The Comparison between the two groups was performed using an unpaired Student’*t*-test while one-way ANOVA was used to calculate *p*-values among multiple groups. Statistical significance was defined as *p* < 0.05 (*p < 0.05, ^**^p < 0.01, ^***^p < 0.001, ^****^p < 0.0001).

Other materials and methods were described in the Supporting Information section.

## Results

### Antimicrobial Effects of Eltrombopag Against *Staphylococcus aureus* Without Resistance Selection

As shown in Table 1, EP exhibited strong antimicrobial activity against Gram-positive strains, with MIC values of 2–8 μg/mL against type strains and clinical isolates of both MRSA and methicillin-sensitive *S. aureus* (MSSA), an MIC of 8 μg/mL against *S. epidermidis*, and an MIC of 2 μg/mL against VAN-resistant *E. faecium.* EP showed bacteriostatic activities against both *S. aureus* and *E. faecium*, with MBC of more than 32 μg/mL, but showed a bactericidal activity against *S. epidermidis*, with an MBC of 16 μg/mL. However, EP showed a relatively weak antimicrobial activity against *E. faecalis* with MICs of more than 32 μg/mL. In addition, the olamine salt form of EP [EP(O)] was also used to assess the antimicrobial efficacy against *S. aureus*. However, the antimicrobial activity of EP was moderately impeded in the presence of olamine (Table 1). Thus, EP was selected for further study.

**TABLE 1:** Antimicrobial susceptibility test of EP and EP(O).

Strain	MIC (μg/ml)				MBC (μg/ml)			
	EP	EP(O)	VAN	OXA	EP	EP(O)	VAN	OXA
***S. aureus***								
ATCC 29213	4	8	1	0.25	> 32	>32	8	16
MW2^a^	8	8	1	64	> 32	>32	2	> 128
ATCC 43300^a^	8	8	1	64	> 32	>32	1	> 128
USA300^a^	4	8	1	64	> 32	>32	4	> 128
SAJ1^a,b^	8	16	4	> 128	>32	> 32	8	>128
SA01	4	8	2	2	> 32	>32	4	4
SA02	4	8	2	0.25	> 32	>32	4	16
SA04	4	4	0.5	0.5	> 32	>32	1	5
SA05	2	4	0.5	0.25	> 32	>32	2	16
SA06	4	4	0.5	0.5	> 32	>32	2	1
SA07	4	4	1	1	> 32	>32	2	4
SA08	4	8	2	4	> 32	>32	4	4
SA0524^a^	4	4	1	> 128	>32	> 32	2	>128
SA2231^a^	4	8	2	> 128	>32	> 32	4	>128
***S. epidermidis***								
RP62A	8	8	2	–	16	> 32	8	–
ATCC 12228	8	8	1	–	16	> 32	4	–
***E. faecalis***								
ATCC 29212	32	> 32	2	–	> 32	>32	> 32	–
***E. faecium***								
U101^c^	2	> 32	>32	–	> 32	>32	> 32	–

*^a^MRSA; ^b^VAN-intermediate strain; ^c^VAN-resistant strain.*

*EP(O): the bisolamine salt of EP; OXA: oxacillin.*

**FIGURE 1:**
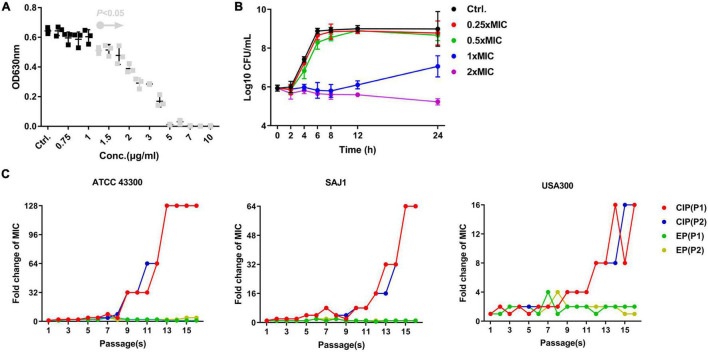
Antimicrobial activity of EP against *S. aureus*. **(A)** Dose-dependent growth inhibition of *S. aureus* USA300 in the presence of varying concentrations of EP. Gray dots indicate *p* < 0.5. **(B)** Bacterial killing kinetics of EP against *S. aureus* USA300. **(C)** Occurrence of spontaneous EP- and ciprofloxacin (CIP)-resistant mutants over 15 days of serial passage in two parallel tests (P1 and P2). The MIC of EP and CIP was determined by CLSI-recommended methods. After overnight culturing, sub-MIC (1/2 × MIC) of bacterial suspension was diluted 1000-fold and used in the further antimicrobial susceptibility testing. Each line indicates a parallel test.

EP exhibited a dose-dependent bacterial growth inhibition activity against *S. aureus* at concentrations greater than 1.5 μg/mL (Figure 1A). Consistent with the growth inhibition activity, the results of the time-killing assay revealed that EP inhibited the increase in CFU counts within 12 h at concentrations greater than 1 × MIC (Figure 1B). Furthermore, the MIC of CIP against *S. aureus* began to increase at days 8 to 10 in the presence of sub-MIC of CIP, but there were no resistant mutants in the presence of sub-MIC of EP for 15 days (Figure 1C). The spontaneous resistance frequencies were also measured at 2 × to 4 × MIC for EP and RFP. EP had low frequency of resistance of ∼10^–9^ at 2 × MIC, and non-detectable frequency of resistance at 4 × MIC. As a positive control, RFP showed a higher frequency of resistance on the order of ∼10^–8^ to ∼10^–9^ at 2 × to 4 × MIC (Table 2).

**TABLE 2:** Single-step resistance selection of EP for *S. aureus* USA300.

Antimicrobial	Spontaneous resistance frequency	
	2 × MIC	4 × MIC
EP	1.49(± 0.95) × 10^–9^	< LOD
RFP	4.97(± 4.64) × 10^–8^	2.85(± 2.00) × 10^–9^

*LOD: limit of detection (∼1 × 10^–10^).*

### Effective Anti-biofilm Effects of Eltrombopag

*Staphylococcus aureus* showed a dose-dependent biofilm-enhancing effect in the presence of increasing concentrations of glucose (Supplementary Figure 1). Thus, we chose TSB with 0.15% GLU (TSB-g) to perform further biofilm-related assays.

**FIGURE 2:**
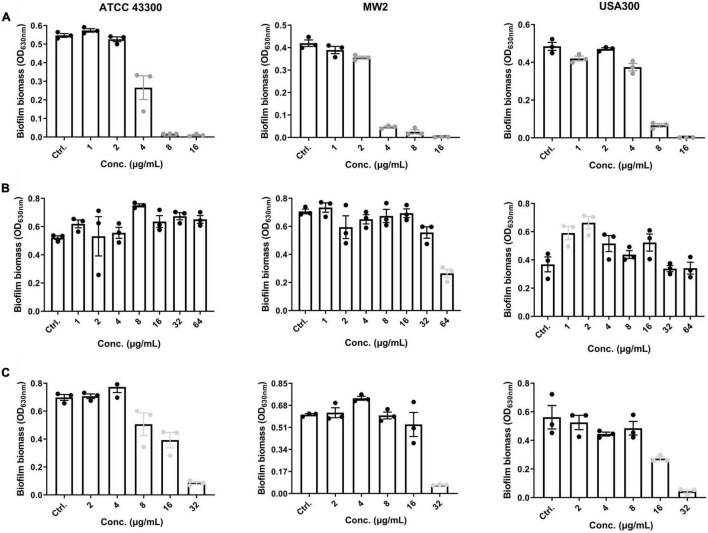
Effective anti-biofilm activity of EP against *S. aureus*. **(A)** Inhibition of biofilm formation by *S. aureus* in the presence of varying concentrations of EP. **(B)** Limited biofilm eradication effects of VAN even at high concentrations. **(C)** Eradication of 24-h mature biofilms in the presence of the indicated concentrations of EP. Mature biofilms were formed in 96-well cell culture plates with TSB-g. All experiments were performed in triplicate. Results are represented as mean ± standard deviation. Gray dots indicate *p* < 0.05.

EP was effective in inhibiting *S. aureus* biofilm formation at concentrations of 2–4 μg/mL, which approximate 1 × MIC (Figure 2A). Thus, the effects of EP on the inhibition of biofilm formation are probably because of the bacteriostatic activity of EP. *S. aureus* planktonic cells were sensitive to VAN, with MIC and MBC values of 1–4 μg/mL and 1–8 μg/mL, respectively (Table 1); however, no biofilm eradication efficacy was observed even at 64 μg/mL of VAN (Figure 2B). Surprisingly, EP was able to eradicate the 24-h mature biofilm at concentrations of 8–32 μg/mL (Figure 2C), indicating the presence of another potential mechanism of how EP targets the components or the regulating system involved in *S. aureus* biofilms.

### Eltrombopag Disrupts Proton Motive Force of *Staphylococcus aureus*

**FIGURE 3:**
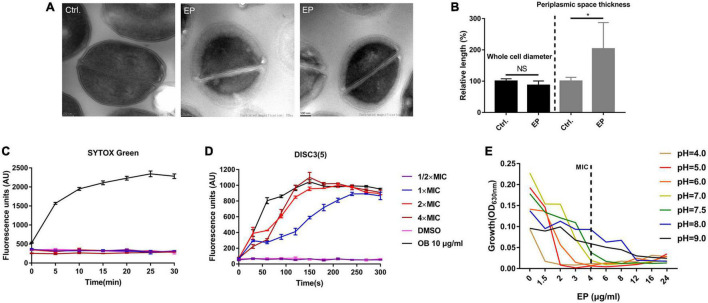
Disruption of proton motive force by EP. **(A)** TEM showed edema formation in EP treated cells. *S. aureus* USA300 was treated with EP at 5 × MIC for 1 h. After washing with phosphate-buffered saline, samples were fixed with 2.5% glutaraldehyde and subject to further TEM observation. **(B)** Relative length of bacterial cell diameters and periplasmic space thickness after treatment with EP. The calculation was based on TEM observations. **(C)** Bacterial cell membrane integrity detection by SYTOX Green staining. OB (otilonium bromide, 10 μg/mL) was used as the positive control. **(D)** Effects of EP on bacterial cell membrane potential stability by DiSC3(5) staining. OB (10 μg/mL) was used as the positive control. **(E)** Growth inhibition by EP against *S. aureus* USA300 in pH-adjusted MH broth. Shown is the mean of three biological replicates.

Transmission electron microscopy (TEM) was used to observe the structure changes by EP treatment. By TEM, the apparent nucleoid aggregation and swelling in the cytoplasm of *S. aureus* cells were observed after 1 h of treatment with 5 × MIC of EP (Figure 3A). However, no changes were observed in the cell diameter (Figure 3B). Further, SYTOX Green, a cell membrane-sensitive fluorescent dye, was used to further detect the effect of EP on cell membranes. However, no cell membrane-disrupting activity was detected in the presence of various concentrations of EP (Figure 3C). DiSC3(5) is a hydrogen/potassium ion-sensitive dye that can also act as an indicator of the PMF (Liu et al., 2020). The fluorescence intensity was significantly enhanced by EP at a concentration of 1 × MIC (Figure 3D), indicating that the action mechanisms of EP could be mediated by weakening the PMF. In addition, as the PMF was driven by the concentration of H + /K + in cells, by adjusting the culture medium to various pH values, the antimicrobial effects of EP improved as the concentration of hydrogen ions increased (Figure 3E).

### Gene Dysregulation of *Staphylococcus aureus* by Eltrombopag Treatment

**FIGURE 4:**
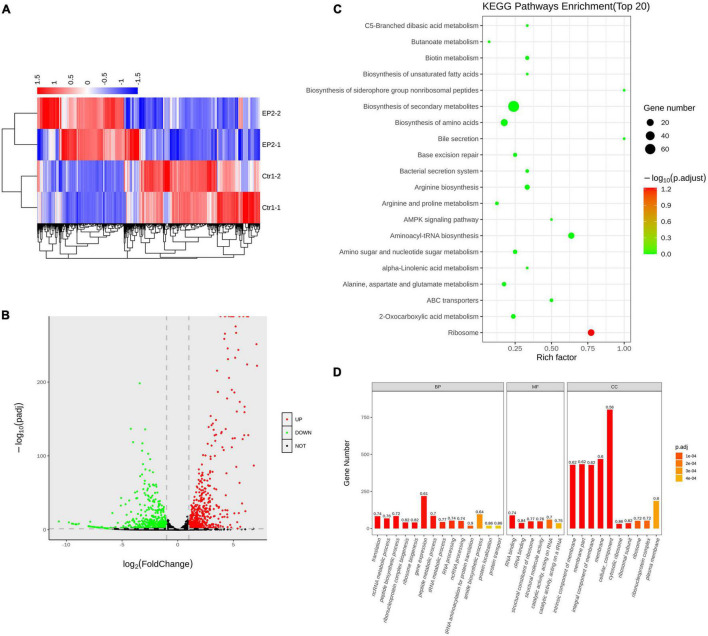
Transcriptome analysis of *S. aureus* USA300 after treatment with 5 × minimum inhibitory concentration of EP for 1 h. **(A)** Heat-map of differentially expressed genes. **(B)** Upregulated and downregulated genes are shown by red and green dots, respectively. **(C)** Bubble chart of top 20 enrichment genes in KEGG pathways. Dot color indicates the value of adjusted *p*, and dot size indicates the number of enrichment genes in each pathway. **(D)** Gene ontology analysis of relative gene expression by whole transcriptome sequencing. Genes were enriched using the cellular component (CC), biological process (BP), and molecular function (MF).

By performing whole gene transcriptome analysis, we found that EP significantly changed the gene expression of *S. aureus* USA300 after 1 h of treatment in the presence of 5 × MIC of EP. As described in the heat map (Figure 4A) and volcano plots (Figure 4B), a total of 1065 differently expressed genes were statistically significant with Padj < 0.05. Among which, 536 genes were upregulated and 529 genes were downregulated. KEGG pathway enrichment analysis revealed that these significantly affected genes were widely involved in the biosynthesis of secondary metabolites and amino acids associated with aminoacyl-tRNA-related pathways and ribosome-related pathways (Figure 4C). Gene ontology analysis revealed that although EP had a wide range of effects on multiple function-related genes, the upregulated and downregulated genes were mainly enriched in cellular components and cell membranes (Figure 4D).

### Eltrombopag in Combination With Vancomycin Shows Synergistic Antimicrobial Activity Against Methicillin-Resistant *Staphylococcus aureus in vitro*

**FIGURE 5:**
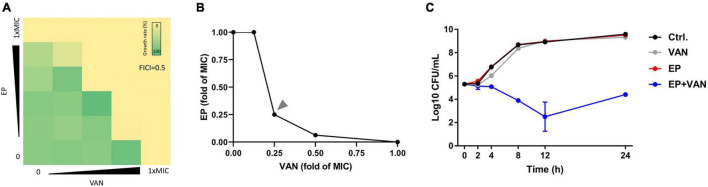
Synergistic antimicrobial activity between EP and vancomycin. **(A)** The synergistic antimicrobial efficacy between EP and VAN by checkerboard dilution assay. FICI, fractional inhibitory concentration index. **(B)** Quantification of panel **(A)** by calculating the fold change of MIC. **(C)** *S. aureus* killing kinetics of sub-MIC of EP (1 μg/mL) alone or in combination with sub-MIC of VAN (1 μg/mL).

To reduce the side effects and enhance the antimicrobial activity of EP, we tried to combine EP with VAN. By checkerboard dilution assay, we found the significant synergistic antimicrobial activity between EP and VAN with FICI = 0.5 (Figures 5A,B). Further, by time-killing assay, we found that single use of sub-MIC EP or VAN exhibited no bactericidal activity against *S. aureus*, however, obvious synergistic bactericidal effect was shown when used in combination (Figure 5C).

### *In vivo* Antimicrobial Effects of Eltrombopag Against Methicillin-Resistant *Staphylococcus aureus* Infections

**FIGURE 6:**
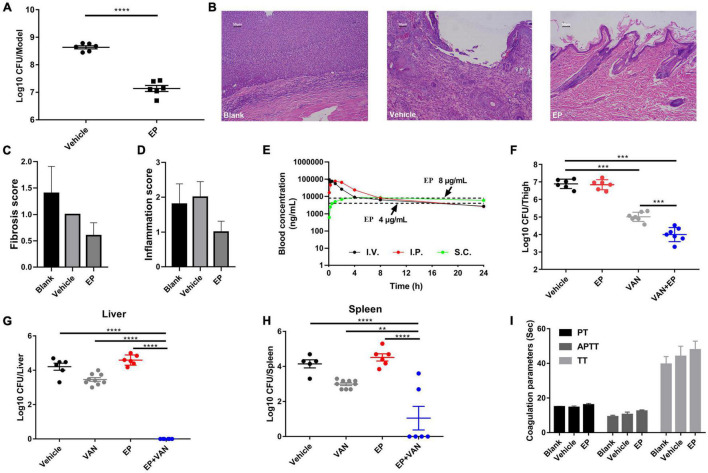
*In vivo* efficacy of EP in mouse infection models. **(A)** Acute skin infection model. Each group of *S. aureus* USA300-infected BABL/C mice was treated with 2% (w/v) EP or vehicle (ointment) every 5 h for a total of 24 h. The infected skin tissues were excised and homogenized 5 h after the last treatment dose and subjected to CFU counting. **(B)** H&E staining of New Zealand rabbit skin after treatment with EP or vehicle (ointment) for 7 days. The backs of adult New Zealand rabbits were shaved with a razor and scratched with an abrasive paper to form a wound measuring 2 cm × 2 cm. Each group was treated with EP (2%, w/v), blank (saline), or vehicle (ointment) every 5 h on the first day and every 12 h in the succeeding days. On the last day of treatment, the infected skin tissues were excised and stained with H&E. **(C)** Fibrosis and **(D)** inflammation scores of the skin tissues were measured by H&E staining. **(E)** Pharmacokinetic curves of EP (30 mg/kg) administered *via* i.v., i.p., and s.c. injection. Dotted lines indicate the concentrations of EP that reached the minimal inhibitory concentration (MICs) (4 and 8 μg/mL) in serum. **(F)** Antimicrobial activity of EP alone or in combination with VAN in a deep-thigh mouse infection model. Six to seven neutropenic ICR mice (biologically independent animals) per group were treated with 30 mg/kg of EP (i.p.) alone or in combination with 30 mg/kg of VAN (i.p.) or vehicle (5% Kolliphor + 5% ethanol, i.p.). EP and VAN were administered at intervals of 12 h and 5 h, respectively, for a total of 24 h. After the last treatment dose, the infected thigh tissues were excised and homogenized and subjected to CFU counting. **(G,H)** Effect of EP alone or in combination with VAN on microbial burden in liver and spleen in a mouse model of intra-abdominal infection. Mice were infected with 0.5 mL of the indicated inoculum of *S. aureus* USA300 (i.p.) and 5% (w/v) mucin. Each group was treated with 30 mg/kg of VAN (i.p.) alone or in combination with 30 mg/kg of EP (i.p.) at intervals of 5 h and 12 h, respectively, for a total of 24 h. After the last dose, the microbial burden in the liver and spleen was assessed by CFU counting. **(I)** Plasma coagulation parameters (PT, APTT, and TT) of the rats after treatment with 30 mg/kg of EP (i.p.), vehicle (i.p.), or blank (i.p.), two doses on the first day and one dose in 3 succeeding days.

**FIGURE 7:**
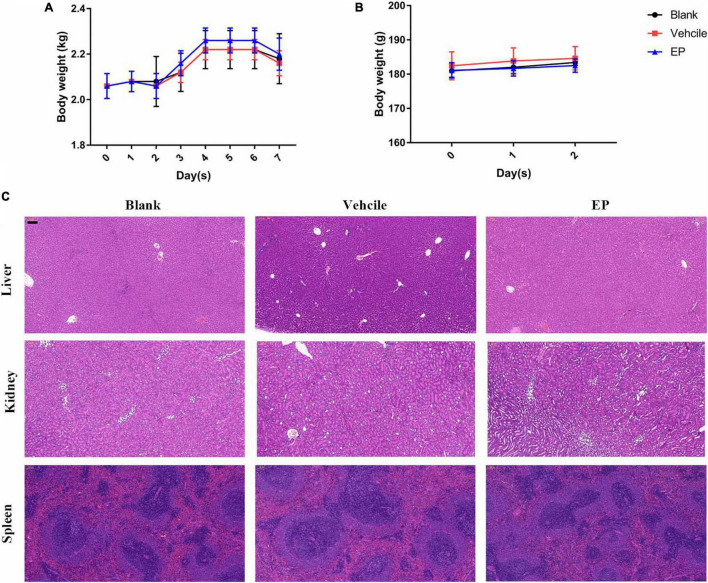
*In vivo* toxicity of EP. **(A)** Body weights of New Zealand rabbits during EP treatment in a skin infection model for seven days. **(B)** Body weights of rats after treatment with 15 mg/kg of EP (I.P.) for 3 consecutive days. **(C)** No signs of hepatic, renal, or splenic toxicity were observed in rats by H&E staining. Six rats per group were treated with blank (saline, I.P.), control (5% Kolliphor + 5% ethanol, I.P.), or EP (15 mg/kg, I.P.) every 12 h for a total of 3 days. After the last treatment dose, blood was collected, and the mice were euthanized. The organs were excised and stained with H&E stain.

Prior to *in vivo* evaluation, the cytotoxicity of EP against mammalian cells was determined by performing the hemolysis assay using the CCK-8 kit. No hemolysis was observed in the presence of EP even at the highest concentration of 64 μg/mL (Supplementary Figure 2A). Similarly, EP at the indicated concentrations showed no toxicity in human cancer cells or normal cells, including adenocarcinoma A549 alveolar basal epithelial cells (Supplementary Figure 2B), human peripheral blood leukemic Jurkat T cells (Supplementary Figure 2C), human embryonic kidney NEK-293T cells (Supplementary Figure 2D), and human non-small cell lung cancer NCI-H23 cells (Supplementary Figure 2E). Furthermore, an acute *S. aureus* wound infection model was used to assess the antimicrobial effects of EP in topical use. Treatment with EP (2% w/v) every 5 h for a total of 24 h significantly reduced the bacterial burden in infected skin from 8.64 to 7.14 log10 CFU per model (Figure 6A). To examine the toxicity of EP in the skin, the back of each of the 15 adult New Zealand rabbits was shaved, and a wound measuring 2 cm × 2 cm was made on the back using an abrasive paper. The wounds were treated with two doses of 2% EP every 4 h on the first day, followed by one dose per day for 7 days. Then, hematoxylin-eosin (H&E) staining, coagulation parameters, and cardiac/hepatonephric/hematological biomarkers were detected to determine the *in vivo* toxicity of EP. After the first day of treatment, no statistical differences were found between EP-treated and control groups (blank and vehicle) in terms of the coagulation parameters such as activated partial thromboplastin time (APTT), prothrombin time (PT), thrombin time (TT) (Supplementary Table 2), and biomarkers for liver [alanine transaminase (ALT)], kidney [blood urea nitrogen (BUN)], and cardiac [creatinine kinase-MB (CK-MB)] (Supplementary Table 3), and the routine blood parameters [e.g., leukocyte classification and platelet (PLT)/reticulocyte (RET) count] (Supplementary Tables 4, 5). Similarly, after 7 days of treatment, no statistical differences were found among the groups in terms of these parameters (Supplementary Tables 6–9). No redness, swelling, and edema were observed in the three groups by light microscopy with H&E staining. Moreover, no changes in body weight were observed in the groups (Figure 7A). After 7 days, the control groups showed inflammatory cell infiltration in the dermis and subcutaneous tissue, proliferation of fibrous connective tissue, and absence of local epidermal tissue formation. In contrast, the EP-treated group showed minimal inflammatory cell infiltration in the dermis and subcutaneous tissue, proliferation of fibrous connective tissue, and presence of new epidermal tissue formation (Figure 6B). Statistical analysis revealed that fibrosis and inflammation scores had a tendency to decrease in the EP-treated group (Figures 6C,D). Thus, in addition to having an antimicrobial activity, EP might have an anti-inflammatory activity that can inhibit inflammation and fibrosis and improve wound repair.

Determination of pharmacokinetics (PK) of EP involved the use of a single dose of 30 mg/kg of EP administered *via* intravenous (i.v.), i.p., and subcutaneous (s.c.) injections. The PK of EP was dose-dependent and linear. The mean C_max_ values were 93.90, 78.88, and 9.48 μg/mL at approximately 0.08, 0.83, and 5.33 h after dosing, respectively, for of i.v., i.p., and s.c. administrations, respectively. The area under curve (AUC_0–t_) values were 253.29, 368.04, and 174.51 μg⋅h/mL for i.v., i.p., and s.c. administrations, respectively. The mean half-life (T_1/2_) was more than 16.06 h for all doses. T ≥ MIC (4 μg/mL) was more than 16 h on average (Figure 6E and Supplementary Table 1). Accordingly, we selected a dose of 30 mg/kg and an interval of 12 h for further evaluation using systemic infection models. In a deep-thigh infection model, a single treatment with EP showed no reduction in bacterial burden; however, the addition of VAN effectively enhanced the antimicrobial activity of EP *in vivo*, with a 2.89-fold Δlog10 CFU reduction per thigh compared with the log10 CFU in the vehicle group (Figure 6F). Similarly, the combination of EP and VAN showed an *in vivo* synergistic antimicrobial activity in an *S. aureus* peritonitis-sepsis infection model. Two doses of EP combined with five doses of VAN administered within 24 h effectively decreased the bacterial burden by 4.21- and 3.01-fold Δlog10 CFU in mouse liver and spleen, respectively, compared with the log10 CFU in the vehicle group (Figures 6G,H). To assess the *in vivo* toxicity of EP in the systemic infection model, SD rats were treated with EP every 12 h, lasting for a total of 3 days. As expected, no changes in body weight (Figure 7B) and coagulation parameters (TT, PT, and APTT) among the EP-treated group and the control groups (blank and vehicle) were observed during and after the treatment process (Figure 6I). H&E staining showed that high doses of EP exposure did not result in any pathological changes in the liver, kidney, and spleen of the SD rats (Figure 7C). Moreover, no significant changes in the biomarkers (ALT, BUN, and CK) (Supplementary Table 10), red blood cell (RBC)- and PLT-related parameters (Supplementary Table 11), or leukocyte classification (Supplementary Table 12) were observed. Thus, EP is highly likely to be safe for the treatment of systemic infections.

## Discussion

In the present study, the TpoR agonist EP was found to be an effective antimicrobial agent against MRSA strains *in vitro* and *in vivo* probably by disrupting the balance of PMF in MRSA.

EP has been widely studied by repurposing for its potential as a broad-spectrum anti-cancer and anti-viral drug. In particular, EP was found to inhibit the proliferation of many leukemia cell lines, with IC50 values ranging from 0.56 to 21 μg/mL even in the presence of cytokines, such as granulocyte colony-stimulating factor (G-CSF), erythropoietin or Tpo. The anti-leukemia activity of EP was independent of the TpoR agonist effect (Erickson-Miller et al., 2010). EP was also shown to inhibit the proliferation of hepatocellular carcinoma cell lines at concentrations of 40–100 μg/mL by modulating intracellular iron content (Kurokawa et al., 2015). In addition, through high-throughput screening of FDA-approved drugs, Yuan et al. (2019) were able to identify EP as an effective antiviral agent against thrombocytopenia syndrome virus, with an IC50 of 4.1 ± 0.2 μmol/L. In the present study, EP exhibited an antibacterial activity at relatively low concentrations, with MIC values of 4–8 μg/mL. As reported by Yuan et al. (2019), the 50% cytotoxic concentration (CC50) of EP was 18.4 ± 0.2 μmol/L in Vero cells after a 72 h-incubation and detected by the CellTiterGlo luminescent assay. However, in our study, EP was not cytotoxic to all the test cell lines within 12–24 h even at high concentrations of up to 32 μg/mL by CCK-8 assay. The differences of the studies could be due to the different cell lines, different incubation time and different detecting methods. Similarly to our results, EP was reported to enhance TpoR transfected IRF-1 cells relative gene expression even at the concentration of 50 μM (Erickson-Miller et al., 2009). EP could also prevent cell death at the tested concentraions (Spitz et al., 2021).

PMF disruptors were found to have the ability to reduce or reverse antibiotic resistance and the spread of virulence factors (Jeon et al., 2019; Domenech et al., 2020; Liu et al., 2020). In our study, we used the HCl/NaOH-adjusted MH broth to change the ΔpH gradients. The antimicrobial activity of EP improved as the extracellular hydrogen ion concentration increased and weakened as the extracellular hydrogen ion concentration decreased (Figure 3E). Furthermore, the change in ΔΨ due to EP was confirmed by DiSC3(5) staining (Figure 3D). Thus, EP is a potential PMF disruptor against MDR *S. aureus*.

The low toxicity of EP makes EP a potential antibiotic for clinical use. All treatments with EP were generally well tolerated, and no serious adverse events occurred (Aslanis et al., 2018), although headache, gastrointestinal symptons, nasopharyngitis/upper respiratory tract infections are the most common reported adverse effects (Merli et al., 2015). In a phase III multicenter study, EP given at 25–75 mg once a day was safe even for long-term use (eight weeks) (Yang et al., 2017). In an open-label EXTEND study by Wong et al. (2017), EP was effective in maintaining platelet counts in patients with ITP, and no obvious adverse effects, such as thrombosis, bone marrow fibrosis, and hepatobiliary dysfunction, occurred after the treatment within a median duration of 2.73 years (2–8.76 years). As a TpoR agonist, EP requires approximately 2 weeks to increase the platelet count to more than 50 × 10^9^/L in patients with ITP (Wong et al., 2017). However, by repurposing EP as an antimicrobial agent against *S. aureus*, the period of treatment would last less than 2 weeks and would not require long-term use. In the present study, EP showed extremely limited toxicity in a murine model *in vivo* for short-term use.

In conclusion, we found that EP showed an effective antimicrobial activity against MDR *S. aureus in vitro* and *in vivo*. EP also eradicated the preformed MRSA biofilms. The underlying antimicrobial mechanisms of EP likely involve PMF disruption. EP has the potential to treat MDR *S. aureus* infections when therapy with conventional antibiotics fails.

## Data Availability Statement

The original contributions presented in the study are included in the article/Supplementary Material, further inquiries can be directed to the corresponding author.

## Ethics Statement

The animal study was reviewed and approved by the Third Xiangya Hospital of Central South University.

## Author Contributions

PS and YW conceived and designed the experiments and supervised the entire study. PS, ShiL, and LZ performed most of the experiments and composed the manuscript. PS, YaL, LX, and ZH analyzed and plotted the results. YiL, ZL, and ShaL provided some methods needed for this research. All authors contributed to the article and approved the submitted version.

## Conflict of Interest

The authors declare that the research was conducted in the absence of any commercial or financial relationships that could be construed as a potential conflict of interest.

## Publisher’s Note

All claims expressed in this article are solely those of the authors and do not necessarily represent those of their affiliated organizations, or those of the publisher, the editors and the reviewers. Any product that may be evaluated in this article, or claim that may be made by its manufacturer, is not guaranteed or endorsed by the publisher.
