# Supplementary material for: Repurposing Eltrombopag as an Antimicrobial Agent Against Methicillin-Resistant Staphylococcus aureus
Source: Front Microbiol. 2022 Jan 24;12:790686. doi: 10.3389/fmicb.2021.790686 (PMC8819062; doi:10.3389/fmicb.2021.790686)
Supplement: Supplementary file 1 [file Data_Sheet_1.docx]

**Supporting Information**

**Supplementary Methods**

**1. Dose-dependent Growth Inhibition Assay**

The assay was performed as previously reported by Carcamo-Noriega *et al*. (Carcamo-Noriega et al., 2019) with slight modifications. In short, the *S. aureus* cultured in the mid-log phase was diluted with PBS broth to 0.5 McFarland standard, and then re-diluted with TSB broth containing various concentrations of EP (0 to 10 μg/mL) at the ratio of 1 : 200. The bacterial suspension was then incubated at 37 ℃ at 150 rpm for 16 h, and the growth was determined by the optical density of 630 nm (OD630). All experiments were repeated in triplicate.

**2. Effects of GLU on S. aureus Biofilm Formation**

We diluted the overnight culture of *S. aureus* with TSB broth supplemented with various concentrations of glucose (0~2.5%). Then added 200 μL of the diluted bacterial suspension to each well of a 96-well cell culture plate. After incubation at 37°C for 24 h, washed 3 times with PBS to remove planktonic cells, the remaining adhered biofilm was air-dried and the optical density at 630 nm (OD_630_) was measured (You *et al*., 2014). All experiments were repeated in triplicate.

**3. Transmission electron microscope (TEM)**

The *S. aureus* USA300 was cultured overnight and then sub-cultured to mid-log phase with fresh TSB broth. The suspension was diluted with EP (5×MIC, 20 μg/mL) in a 50 mL centrifuge tube to a final concentration of ~1×10^6^ CFU/mL, while the suspension containing 0.13% DMSO was used as control. After incubation for 1 h at 37°C and 150 rpm, the tube was centrifuged at 4000 g for 15 min, and the bacterial precipitate was stored in 2.5% glutaraldehyde phosphate buffer. The specimens were then observed by TEM (HITACHI, Tokyo, Japan), and more than five fields of view were randomly recorded for each sample.

**4. Transcriptome Sequencing of *S. aureus***

The *S. aureus* USA300 cells were cultured overnight at 37 ℃ and 150 rpm, diluted with fresh TSB (1:10,000). After incubation to mid-log phase (~ 1×10^6^ - 1×10^7^ CFU/mL), EP was added to the final concentration of 5 × MIC (20 μg/mL) and further incubated at 37 ℃ and 150 rpm for 1 h and cells with 0.13% DMSO were used as control. After incubation, the cells were collected by centrifugation at 1,2000 × g for 2 min at 4 ℃. Total RNA was purified by RNAprep pure Cell/Bacteria Kit (TianGen Biotech. Beijing, China), then rRNA was removed, and Illumina cDNA library was generated using an improved version RNAtag-seq protocol, as reported by Shishkin *et al*. (Shishkin *et al*., 2015). In short, the mRNA was randomly fragmented in fragmentation buffer and reverse transcripted to the fist cDNA strand in the presence of reverse transcriptase and six random hexamers. The second strand was synthesized by adding buffer, dNTPs, and DNA polymerase I, and further purified with AMPure XP beads. The cDNA library was sequenced on the Illumina NextSeq 500 platform to generate paired-end reads, and DESeq2 was used for differential expression analysis. The cluster-gram function in MATLAB 2016a was used to analyze the hierarchical clustering of differentially expressed genes and referred to the database of <http://www.geneontology.org/> for Gene Ontology (GO) annotation analysis. The transcript cluster enrichment analysis was performed by EcoCyc Pathway Tools and referenced the database at https://www.genome.jp/kegg/pathway.html. The assay was set as two biological replicates. The *P* values was calculated by Fisher’s exact test, and padj < 0.05 or |log2foldchange|>1 was defined as statistical significance.

**5. RBC Hemolysis Assay**

Human RBC (hRBC) was purchased from the Hemo Pharmaceutical & Biological Co. (Shanghai, China). Then 150 μL RBC at a concentration of 10% (v/v) were mixed with an equal volume of drugs ranging from 0 - 64 μg/mL. The hRBCs treated with 0.2% Triton X-100 and 0.42 % DMSO were used as a positive control and negative control, respectively. After incubation at 37℃ for 1 h, aspirated the supernatant to measure the A at 540 nm (A540). The experiment was conducted triplicate. The hemolysis rate was calculated according to the following formula (Zhou *et al*., 2020):

Viability (%) =$\text{100×}\text{（}\text{1-}\frac{\text{ }\text{A}_{\text{042\%DMSO}}\text{ - }\text{A}_{\text{sample}}}{\text{A}_{\text{0.42\%DMSO}}}\text{）}$

**6. Mammalian Cells Cytotoxity by EP**

The cell lines of HEK-293T, A549, HCoEpiC, Jurkat, NCI-H23 and BEL-7404 were cultured in the DMEM culture media (in the presence of 10% Gibico FBS with 200 U/mL penicillin-streptomycin) and 1640 culture media, respectively. The cell cultures were incubated in a humidified incubator at 37 ℃ with 5% CO_2_. For the cytotoxicity test, cells in the mid-log phased were trypsinized and diluted with the designated concentration of EP (0 - 32 μg/mL) or 0.2% DMSO (control) in the corresponding medium at the final concentration of 5 × 10^3^ cells per well for 100 μL per well in a 96-well plate. After incubation at 37 ℃ with 5% CO_2_ for 12 and 24 h, 100 μL CCK-8 was added to each well. After another 4 h incubation, the absorbance at 450 nm (A450) was detected (Kim *et al*., 2018). The experiment was conducted triplicate.

**7. *In vivo* Safety Assessment**

**7.1 Dermal Toxicity Study.** The study was approved by the Animal Care and Use committee of the Third Xiangya Hospital of Central South University (No. 2019sydw0211). Fifteen male New Zealand white rabbits (~ 2.05 kg) were purchased from Hunan SJA Experimental Animal Co. Ltd. (Changsha, China). The shaved back skin was scratched with sandpaper to form a 2 cm × 2 cm wound. The ointment containing 2% EP was evenly applied to the whole artificial wound, twice daily, and covered with sterile gauze pad for the first time, with an interval of 12 h. The ointment and physiological saline were used as vehicle and the blank group respectively. The skin was then washed with PBS after 24 h of treatment. In order to determine the systemic toxicity of EP after being absorbed by the skin, the clinical observations, coagulation parameters, blood routine, and multi-organ function biomarkers were determined by CA-530 automatic blood coagulation analyzer (Sysmex, Shanghai, China), BC-5000vet hematology analyzer (Mindray, Shenzheng, China), and Labospect 003 automatic biochemical analyzer (Hitachi, Japan), respectively. And the skin was treated with EP containing ointment once for the next 7 days. The bodyweight was daily recorded before the treatment. Twenty-four hours after the last treatment, clinical observations, coagulation parameters, blood routine, and multiple organ function biomarkers were tested again. The rabbits were sacrificed humanely by intravenous injection of sustained-release agent, the wound skin was excised and stained by H&E, macroscopic examination and inflammation and fibrosis scoring were performed (Breij *et al*., 2018).

**7.2 Systemic Toxicity Study.** Since more biomarkers were observed by taking more blood, the adult male SD rats (body weight: ~ 180 g) were selected instead of mice for systemic toxicity test and the EP dose used by SD rats was 15 mg kg^-1^ which was calculated by [conversion factor × dose of mice (30 mg/kg)]. SD rats were injected (i.p.) with 15 mg/kg of EP, 5% Killophor + 5% ethanol (vehicle), and PBS (blank) respectively for 6 rats per group. The rats were treated with two doses at 12-h intervals on the first day, and once daily for the next 3 days. The bodyweight was recorded daily before treatment. Twenty-four hours after the last dose, blood and organs (liver, kidney, and spleen) were collected and the clinical observations, coagulation parameters, blood routine, and multi-organ functional biomarkers were all tested by the above mentioned machines . The organs were fixed, sectioned and further stained by H&E (Bhando *et al*., 2020).

**8. Pharmacokinetics Analysis.** The 6-8 week old female ICR mice was administered by i.p., i.v., and s.c. treatment with a single dose of 30 mg kg^-1^ of EP. The blood from the jugular vein was collected in an EP tube containing EDTA-2K at 0.083, 0.25, 0.5, 1, 2, 4, 8, and 24 h after the treatment. The plasma was collected after centrifugated at 4 ℃ 6800 × g for 6 min and stored at - 80 ℃. The plasma EP concentration was measured by LC-MS/MS-04 (TQ4000, JEOL, Japan). The parameters of AUC_0-t_, AUC_0-∞_, MRT_0-∞_, C_max_, T_max_, T_1/2_, mean and standard deviation were calculated by Phoenix WinNonlin 7.0 software (Certara, Princeton, NJ) (Ekins *et al*., 2018).

**References**

Breij, A.D., Riool, M., Cordfunke, R.A., Malanovic, N., Boer, L.D., Koning, R.I., *et al*. (2018) The antimicrobial peptide SAAP-148 combats drug-resistant bacteria and biofilms. *Sci Transl Med* **10:** eaan4044.

Carcamo-Noriega, E.N., Sathyamoorthi, S., Banerjee, S., Gnanamani, E., Mendoza-Trujillo, M., Mata-Espinosa, D., *et al*. (2019) 1,4-Benzoquinone antimicrobial agents against *Staphylococcus aureus* and *Mycobacterium tuberculosis* derived from scorpion venom. *Proc Natl Acad Sci U S A* **116:** 12642-12647.

Ekins, S., Lingerfelt, M.A., Comer, J.E., Freiberg, A.N., Mirsalis, J.C., O'Loughlin, K., *et al*. (2018) Efficacy of Tilorone Dihydrochloride against *Ebola* Virus Infection. *Antimicrob Agents Chemother* **62:** e01711-17.

Shishkin, A.A., Giannoukos, G., Kucukural, A., Ciulla, D., Busby, M., Surka, C., *et al*. (2015) Simultaneous generation of many RNA-seq libraries in a single reaction. *Nat Methods* **12:** 323-325.

You, Y., Xue, T., Cao, L., Zhao, L., Sun, H., and Sun, B. (2014) *Staphylococcus aureus* glucose-induced biofilm accessory proteins, GbaAB, influence biofilm formation in a PIA-dependent manner. *Int J Med Microbiol* **304:** 603-612.

Zhou, L., She, P., Tan, F., Li, S., Zeng, X., Chen, L., *et al*. (2020) Repurposing antispasmodic agent otilonium bromide for treatment of *Staphylococcus aureus* infections. *Front Microbiol* **11**: 1720.

Bhando, T., Bhattacharyya, T., Gaurav, A., Akhter, J., Saini, M., Gupta, V.K., and Srivastava, S.K., *et al*. (2020) Antibacterial properties and *in vivo* efficacy of a novel nitrofuran, IITR06144, against MDR pathogens. *J Antimicrob Chemother* **75**: 418-428.

**Supplementary Figures**

**Figure S1.** **Glucose (GLU) enhances biofilm formation by *S. aureus*.** Strains cultured overnight were diluted with TSB to a final concentration of ~10^6^ CFU/mL in the presence of various concentrations of GLU. After a 24-h incubation, the planktonic supernatant was removed, and optical density at 630 nm (OD630) was detected.

**Figure S2. Cytotoxicity of eltrombopag (EP) against various cell lines.** (A) Measurement of hemolytic activity. Two percent of human blood cells were treated with the indicated concentrations of EP for 1 h at 37 °C. Cells treated with 0.2% DMSO and 2% Triton-X 100 were set as the negative control and positive control (100% hemolysis), respectively. The cell lines (B) A549, (C) Jurkat, (D) NEK-293T, and (E) NCl-H23 were treated with various concentrations of EP or 0.2% DMSO (control group) at 37 °C for 12 h or 24 h. The viability of cells was detected using the CCK-8 kit, and absorbance at 450 nm (A450) was measured.

**Figure S1.**


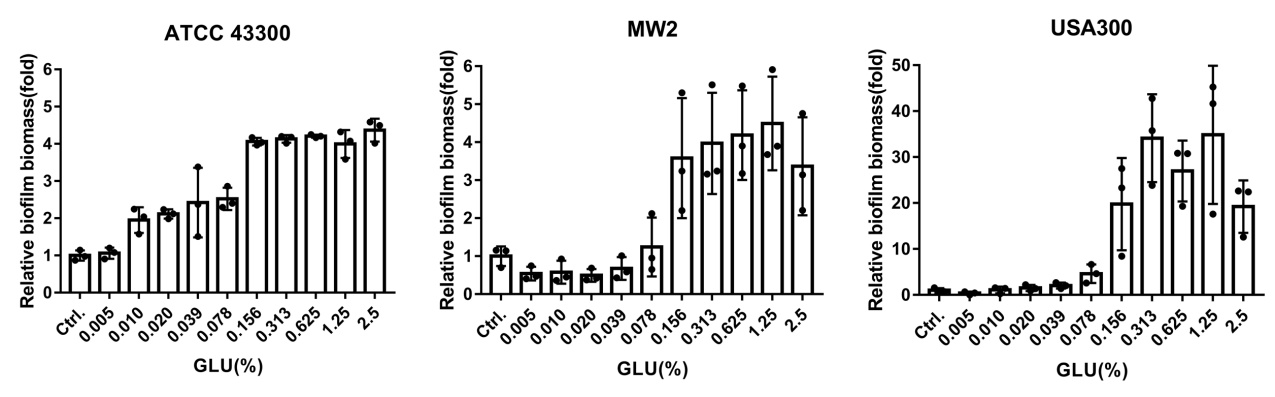


**Figure S2.**

**
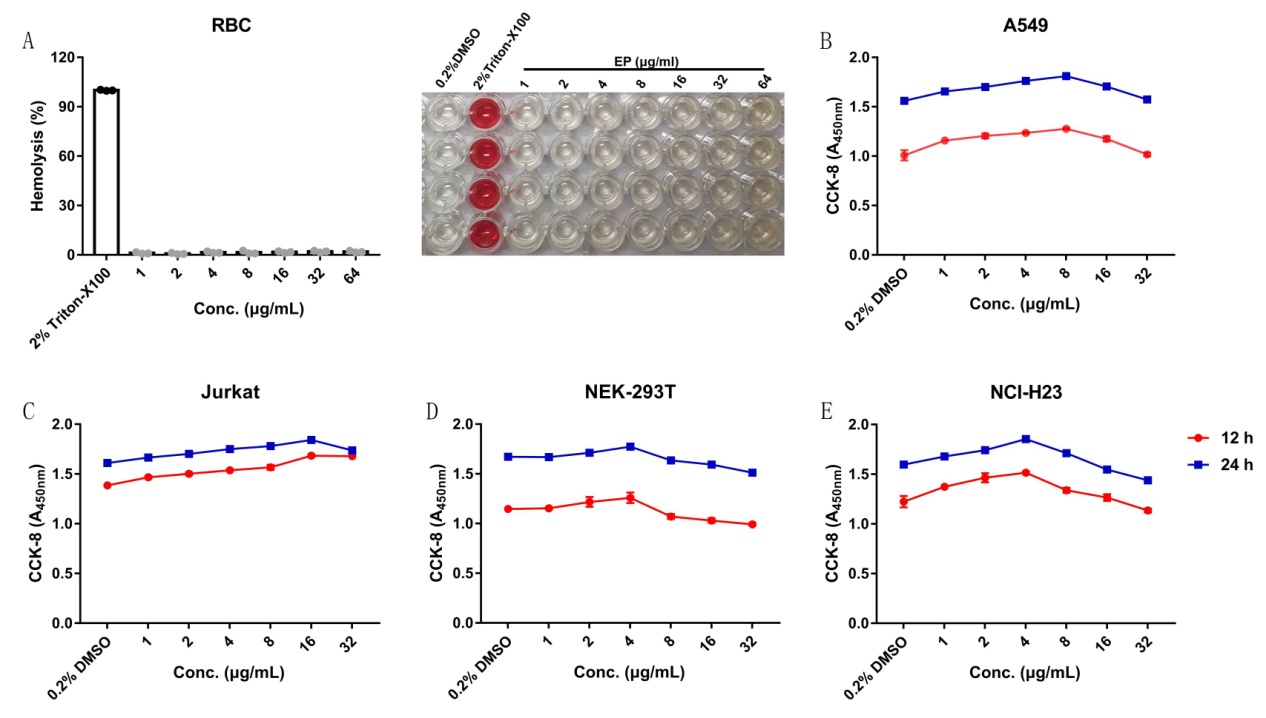
**

**Supplementary Tables**

Table S1. PK parameters of EP

| **Administration Route** | **Dose Level**  **(mg/kg)** | **Caculation** | **T_1/2_**  **(h)** | **T_max_**  **(h)** | **C_max_**  **μg/mL** | **AUC_(0-t)_**  **h*μg/mL** | **AUC_(0-∞）_**  **h*μg/mL** | **MRT_(0-t)_**  **h** | **MRT_(0-∞）_**  **h** | **C_0_(μg/mL)/F(%)** |
| --- | --- | --- | --- | --- | --- | --- | --- | --- | --- | --- |
| IV | 30 | mean | 11.87 | 0.08 | 93.90 | 253.29 | 299.77 | 5.05 | 10.64 | 111.08 |
|  |  | SD | 0.15 | 0.00 | 19.13 | 17.83 | 19.18 | 0.42 | 1.08 | 31.37 |
| IP | 30 | mean | 6.30 | 0.83 | 78.88 | 368.04 | 392.17 | 4.59 | 6.32 | 145.30 |
|  |  | SD | 1.26 | 0.29 | 13.74 | 57.05 | 66.23 | 0.20 | 0.73 | 22.52 |
| SC | 30 | mean | 30.01 | 5.33 | 9.48 | 174.51 | 435.50 | 11.17 | 44.80 | 68.90 |
|  |  | SD | 8.21 | 2.31 | 0.43 | 7.90 | 87.20 | 0.51 | 12.54 | 3.12 |

Table S2. Effects of EP on coagulation function in an acute skin infection model (1 day treatment, N=5)

|  | APTT(Sec) | PT(Sec) | PT(INR) | TT(Sec) |
| --- | --- | --- | --- | --- |
| Blank | 21.97±1.54 | 13.76±0.51 | 1.16±0.04 | 19.00±1.41 |
| Vehicle | 21.71±1.49 | 14.29±0.86 | 1.20±0.07 | 18.15±0.94 |
| EP | 23.14±2.14 | 13.21±1.40 | 1.11±0.12 | 19.95±1.72 |
| *p* (vehicle vs. EP) | 0.425 | 0.238 | 0.243 | 0.145 |

Table S3. Effects of EP on liver, kindey and cardiac function in an acute skin infection model (1 day treatment, N=5)

|  | ALT(U/L) | BUN(mmol/L) | CK-MB(U/L) |
| --- | --- | --- | --- |
| Blank | 47.78±8.04 | 6.976±1.57 | 1599.20±273.02 |
| Vehicle | 53.46±27.41 | 6.742±0.69 | 767.20±281.77 |
| EP | 43.46±11.06 | 6.512±1.18 | 562.40±163.93 |
| *p* (vehicle vs. EP) | 0.654 | 0.951 | 0.412 |

Table S4. Effects of EP on RBC, PLT and its related parameters in an acute skin infection model (1 day treatment, N=5)

|  | RBC  (*10^12^/L) | HGB  (g/L) | PLT  (*10^9^/L) | MPV  (fL) | PDW | PCT  (%) | RET  (per 1000 RBC) |
| --- | --- | --- | --- | --- | --- | --- | --- |
| Blank | 4.69±0.37 | 105.20±6.87 | 363±62.19 | 6.4±0.42 | 6.38±0.81 | 0.23±0.04 | 46.6±4.16 |
| Vehicle | 5.08±0.44 | 107.40±6.66 | 379.4±64.34 | 6.46±0.96 | 6.50±1.06 | 0.24±0.04 | 28.6±8.73 |
| EP | 5.43±0.12 | 115.60±1.67 | 408±68.80 | 6.18±0.47 | 5.98±0.88 | 0.25±0.06 | 30.4±8.56 |
| *p* (vehicle vs. EP) | 0.270 | 0.093 | 0.772 | 0.786 | 0.656 | 0.941 | 0.923 |

Table S5. Effects of EP on leukocyte classification in an acute skin infection model (1 day treatment, N=5)

|  | WBC  (*10^9^/L) | Neu% | Lym% | Mon% | Eos% | Bas% | Neu#  (*10^9^/L) | Lym#  (*10^9^/L) | Mon#  (*10^9^/L) | Eos#  (*10^9^/L) | Bas#  (*10^9^/L) |
| --- | --- | --- | --- | --- | --- | --- | --- | --- | --- | --- | --- |
| Blank | 8.07±2.62 | 39.52±3.50 | 47.62±6.78 | 5.22±3.12 | 5.78±2.65 | 1.86±1.44 | 3.22±1.16 | 3.71±0.69 | 0.47±0.39 | 0.51±0.37 | 0.16±0.17 |
| Vehicle | 8.59±2.32 | 43.84±7.62 | 37.02±9.25 | 7.80±3.99 | 7.50±2.87 | 3.84±2.66 | 3.87±1.65 | 3.19±1.24 | 0.61±0.19 | 0.65±0.37 | 0.28±0.14 |
| EP | 8.11±3.15 | 39.10±7.28 | 39.32±8.89 | 9.34±2.56 | 8.62±3.28 | 3.62±2.65 | 3.26±1.76 | 3.15±1.17 | 0.72±0.20 | 0.67±0.26 | 0.30±0.24 |
| *p* (vehicle vs. EP) | 0.958 | 0.493 | 0.902 | 0.743 | 0.822 | 0.988 | 0.810 | 0.998 | 0.806 | 0.995 | 0.985 |

Table S6. Effects of EP on coagulation function in a 7-day treated skin infection model (N=5)

|  | APTT(Sec) | PT(Sec) | PT(INR) | TT(Sec) |
| --- | --- | --- | --- | --- |
| Blank | 11.7±1.7 | 12.92±0.4 | 1.1±0.0 | 16.33±1.1 |
| Vehicle | 13.7±2.3 | 13.36±1.2 | 1.1±0.1 | 16.63±1.9 |
| EP | 12.2±0.7 | 13.52±0.7 | 1.1±0.1 | 17.39±0.5 |
| *p* (vehicle vs. EP) | 0.374 | 0.951 | >1.000 | 0.636 |

Table S7. Effects of EP on liver, kindey and cardiac function in a 7-day treated skin infection model (N=5)

|  | ALT(U/L) | BUN(mmol/L) | CK-MB(U/L) |
| --- | --- | --- | --- |
| Blank | 48.2±5.8 | 7.91±1.20 | 1733.3±959.7 |
| Vehicle | 36.6±12.5 | 8.64±1.75 | 702.1±460.0 |
| EP | 36.7±6.7 | 8.04±1.11 | 556.0±49.1 |
| *p* (vehicle vs. EP) | 1.000 | 0.776 | 0.926 |

Table S8. Effects of EP on RBC, PLT and its related parameters in a 7-day treated skin infection model (N=5)

|  | RBC  (*10^12^/L) | HGB  (g/L) | PLT  (*10^9^/L) | MPV  (fL) | PDW | PCT  (%) | RET  (per 1000 RBC) |
| --- | --- | --- | --- | --- | --- | --- | --- |
| Blank | 5.05±0.29 | 114±5.86 | 444±156.14 | 6.3±0.40 | 6.9±0.76 | 0.28±0.10 | 71.8±9.50 |
| Vehicle | 4.83±0.21 | 107±3.74 | 588±128.27 | 5.9±0.65 | 6.1±1.02 | 0.34±0.04 | 44.6±19.28 |
| EP | 5.72±0.05 | 122±1.41 | 604±226.28 | 6.2±0.55 | 6.6±0.79 | 0.37±0.13 | 44.6±7.30 |
| *p* (vehicle vs. EP) | <0.001 | <0.001 | 0.989 | 0.667 | 0.642 | 0.879 | >1.000 |

Table S9. Effects of EP on leukocyte classification in a 7-day treated skin infection model (N=5)

|  | WBC  (*10^9^/L) | Neu% | Lym% | Mon% | Eos% | Bas% | Neu#  (*10^9^/L) | Lym#  (*10^9^/L) | Mon#  (*10^9^/L) | Eos#  (*10^9^/L) | Bas#  (*10^9^/L) |
| --- | --- | --- | --- | --- | --- | --- | --- | --- | --- | --- | --- |
| Blank | 8.14±0.82 | 33.9±5.67 | 47.2±12.47 | 6.1±2.49 | 10.7±4.93 | 2.1±2.01 | 2.73±0.28 | 3.92±1.45 | 0.48±0.19 | 0.85±0.35 | 0.16±0.16 |
| Vehicle | 10.81±1.19 | 37.9±8.80 | 39.2±11.10 | 10.0±3.70 | 9.3±3.51 | 3.7±2.51 | 4.18±1.41 | 4.19±1.13 | 1.06±0.37 | 1.00±0.41 | 0.38±0.26 |
| EP | 12.04±3.93 | 46.3±6.33 | 32.4±5.67 | 6.8±2.98 | 12.6±9.90 | 1.9±2.06 | 5.40±1.16 | 3.80±0.92 | 0.83±0.43 | 1.82±1.84 | 0.19±0.17 |
| *p* (vehicle vs. EP) | 0.708 | 0.187 | 0.558 | 0.270 | 0.723 | 0.427 | 0.208 | 0.863 | 0.560 | 0.492 | 0.330 |

Table S10. Effects of EP on liver, kindey and cardiac function in a systemic infection model (N=6)

|  | ALT (U/L) | BUN (mmol/L) | CK (U/L) |
| --- | --- | --- | --- |
| Blank | 32.17±5.46 | 8.52±0.71 | 1618.67±606.88 |
| Vehicle | 28.83±7.03 | 8.36±1.28 | 1239.17±494.85 |
| EP | 28.67±8.50 | 7.63±0.91 | 1434.33±966.82 |
| *p* (vehicle vs. EP) | 1.000 | 0.428 | 0.886 |

Table S11. Effects of EP on RBC, PLT and its related parameters in a systemic infection model (N=6)

|  | RBC  (*10^12^/L) | HGB  (g/L) | PLT  (*10^9^/L) | MPV  (fL) | PDW | PCT  (%) | RET  (%) |
| --- | --- | --- | --- | --- | --- | --- | --- |
| Blank | 7.74±0.77 | 158±13.11 | 1217±104.00 | 5.97±0.29 | 15.32±0.04 | 0.72±0.06 | 0.68±0.14 |
| Vehicle | 7.31±0.34 | 149±5.47 | 1087±103.76 | 5.95±0.10 | 15.30±0.17 | 0.65±0.06 | 0.60±0.09 |
| EP | 7.46±0.31 | 150.83±7.00 | 1057±64.14 | 5.83±0.29 | 15.18±0.21 | 0.62±0.05 | 0.55±0.08 |
| *p* (vehicle vs. EP) | 0.872 | 0.936 | 0.842 | 0.677 | 0.407 | 0.640 | 0.72 |

Table S12. Effects of EP on leukocyte classification in a systemic infection model (N=6)

|  | WBC  (*10^9^/L) | Neu% | Lym% | Mon% | Eos% | Bas% | Neu#  (*10^9^/L) | Lym#  (*10^9^/L) | Mon#  (*10^9^/L) | Eos#  (*10^9^/L) | Bas#  (*10^9^/L) |
| --- | --- | --- | --- | --- | --- | --- | --- | --- | --- | --- | --- |
| Blank | 7.40±2.35 | 9.04±1.62 | 83.02±1.78 | 6.52±2.36 | 1.07±0.45 | 0.38±0.19 | 0.65±0.18 | 6.12±1.83 | 0.52±0.31 | 0.08±0.05 | 0.03±0.02 |
| Vehicle | 6.41±2.43 | 8.03±1.86 | 85.82±1.69 | 4.88±0.56 | 1.05±0.29 | 0.21±0.17 | 0.51±0.15 | 5.51±2.10 | 0.32±0.17 | 0.07±0.02 | 0.01±0.02 |
| EP | 6.44±2.76 | 14.42±9.12 | 78.40±9.07 | 6.02±1.83 | 0.88±0.32 | 0.28±0.08 | 0.83±0.45 | 5.16±2.56 | 0.37±0.18 | 0.06±0.04 | 0.02±0.01 |
| *p* (vehicle vs. EP) | 0.1000 | 0.139 | 0.077 | 0.514 | 0.698 | 0.717 | 0.175 | 0.959 | 0.925 | 0.896 | 0.588 |
